# Planetare Gesundheit – transformative Lehr- und Lernformate zur Klima- und Nachhaltigkeitskrise für Gesundheitsberufe

**DOI:** 10.1007/s00103-021-03289-x

**Published:** 2021-02-15

**Authors:** Katharina Wabnitz, Sophia Galle, Louise Hegge, Oskar Masztalerz, Eva‑Maria Schwienhorst-Stich, Michael Eichinger

**Affiliations:** 1 Institut für Medizinische Informationsverarbeitung, Biometrie und Epidemiologie (IBE), Pettenkofer School of Public Health, Ludwig-Maximilians-Universität, Elisabeth-Winterhalter-Straße 6, 81377 München, Deutschland; 2 The Primary Care Unit, Department of Public Health and Primary Care, University of Cambridge, Cambridge, Großbritannien; 3 Julius-Maximilians-Universität Würzburg, Würzburg, Deutschland; 4 Technische Universität München, München, Deutschland; 5 Deutsche Allianz Klimawandel und Gesundheit (KLUG e. V.), Berlin, Deutschland; 6 Charité – Universitätsmedizin Berlin, Berlin, Deutschland; 7 Zentrum für Studiengangsmanagement und -entwicklung (ZSME), Julius-Maximilians-Universität Würzburg, Würzburg, Deutschland; 8 Mannheimer Institut für Public Health, Sozial- und Präventivmedizin, Medizinische Fakultät Mannheim der Universität Heidelberg, Mannheim, Deutschland; 9 Abteilung für Pädiatrische Epidemiologie, Institut für Medizinische Biometrie, Epidemiologie und Informatik, Universitätsmedizin der Johannes-Gutenberg-Universität Mainz, Mainz, Deutschland; 10 Klinik für Kinder- und Jugendmedizin, Medizinische Fakultät Mannheim der Universität Heidelberg, Mannheim, Deutschland

**Keywords:** Lehre, Gesundheitswesen, Klimawandel, Transformation, Nichtschaden, Education, Health system, Climate change, Transformation, Do no harm

## Abstract

Die Dringlichkeit der Klima- und Nachhaltigkeitskrise und ihre Auswirkungen auf die menschliche Gesundheit rücken auch im deutschen Gesundheitswesen zunehmend in den Fokus. Um ein weiteres Fortschreiten der Klima- und Nachhaltigkeitskrise zu verhindern, sind tiefgreifende Transformationsprozesse in allen gesellschaftlichen Sektoren notwendig (z. B. Verkehr, Energieerzeugung, Ernährungssystem). Angehörige der Gesundheitsberufe haben auf Basis ihrer ethischen Verpflichtung des Nichtschadens und ihrer guten Vertrauenswerte in der Gesellschaft das Potenzial, einen wichtigen Beitrag zu den notwendigen Transformationsprozessen zu leisten.

Damit sie dieses Potenzial zur Gänze ausschöpfen können, sollten Angehörige der Gesundheitsberufe im Rahmen ihrer Aus‑, Fort- und Weiterbildung bei der Entwicklung von Kompetenzen begleitet werden, die zu transformativem Handeln befähigen. Wir führen in diesem Artikel in das Konzept der *planetaren Gesundheit* ein, das hierfür sowohl inhaltliche als auch ethische Orientierung gibt. Außerdem geben wir einen Überblick über aktuelle Lehr- und Lernformate und identifizieren Aspekte, die zur Weiterentwicklung der Lehre im Bereich planetare Gesundheit beitragen könnten.

## Abstract (en)

The urgency of the climate and sustainability crises and their health effects are receiving increasing attention in the German health system. To avoid further exacerbation of these crises, profound transformative processes in all sectors of society are needed (e.g. transport, energy production, and food systems). Based on the ethical imperative of non-maleficence and the high levels of trust in society, health professionals have great potential to make an important contribution to the necessary transformative processes.

In order to fully harness this potential, health professionals should be supported in developing competencies to take transformative action during their pre- and postgraduate education and professional training. In this article, we introduce the concept of *planetary health*, as this concept provides orientation for this both ethically and with regards to the subject matter. Furthermore, we provide an overview of current teaching and learning formats and identify aspects that could contribute to further developing planetary health education.

## Bedeutung der Klima- und Nachhaltigkeitskrise für die Gesundheit

Die Klimakrise stellt die größte Bedrohung der menschlichen Gesundheit im 21. Jahrhundert dar [1]. Die mit ihr einhergehenden Umweltveränderungen, die unter anderem zu Hitzewellen, Überflutungen und Unterernährung durch Ernährungsunsicherheit führen, wirken sich bereits heute negativ auf die Gesundheit einer großen Anzahl von Menschen aus [2]. Dabei treten diese Auswirkungen nicht nur in besonders vulnerablen Regionen des globalen Südens, sondern auch in Ländern wie Deutschland immer deutlicher zutage [3]. Zudem zeigt die gegenwärtige COVID-19-Pandemie eindrücklich, dass neben Klimaveränderungen beispielsweise auch der Verlust an Biodiversität und natürlichen Habitaten – zum Teil durch Wildtierhandel verschärft – eng mit der menschlichen Gesundheit zusammenhängen kann [4]. Werden durch diese globalen Umweltveränderungen sogenannte planetare Belastungsgrenzen überschritten (Infobox 1), steigt das Risiko, dass nicht-lineare Veränderungsprozesse und sich selbst verstärkende Feedbackmechanismen einsetzen, die durch menschliches Handeln nicht mehr aufzuhalten sind [5].

Während die Dringlichkeit der Klima- und Nachhaltigkeitskrise und ihre gesundheitlichen Auswirkungen im deutschen Gesundheitswesen bis vor Kurzem nur am Rande berücksichtigt wurden, rückt das Thema nun zunehmend in den Fokus. Dies zeigt sich nicht zuletzt an der Forderung des Deutschen Ärztetags 2019 nach einer Fokussierung auf die Klimakrise [6]. Auch auf gesundheitspolitischer Ebene wird der Klima- und Nachhaltigkeitskrise vermehrt Beachtung geschenkt, indem u. a. im Bundesministerium für Gesundheit ein Referat geschaffen wurde, das sich v. a. mit den gesundheitlichen Auswirkungen der Klima- und Nachhaltigkeitskrise beschäftigt [7].

## Lehre und Transformation

Um den skizzierten Herausforderungen für die menschliche Gesundheit zu begegnen, muss die Aus‑, Fort- und Weiterbildung von Fachkräften im Gesundheitswesen rasch und flächendeckend um Lehr- und Lernformate zur Klima- und Nachhaltigkeitskrise ergänzt werden [8]. Neben der Vorbereitung auf die Behandlung klimabedingter Gesundheitsprobleme, wie beispielsweise Hitzeschäden oder Dehydratation, sollten diese Lehr- und Lernformate insbesondere zu transformativem Handeln befähigen [8, 9].

Transformation meint die umfassende Veränderung aller gesellschaftlichen Strukturen und Sektoren, die aktuell die planetare Gesundheit bedrohen [10]. Dies schließt technische Lösungen wie die Elektromobilität oder die Energieerzeugung durch erneuerbare Energien, aber auch nachhaltige Verhaltensweisen bezüglich Konsum und Ernährung ein. Insbesondere braucht es zum Schutz der Gesundheit von Menschen heute und in der Zukunft eine Transformation unserer Werte und Haltungen, da diese wesentliche Bedingungsfaktoren für Veränderungen unseres Politik- und Wirtschaftssystems darstellen [11].

### Planetare Gesundheit

Das Konzept der planetaren Gesundheit (*planetary health*) kann für die Gestaltung und Umsetzung gesellschaftlicher Transformationsprozesse Orientierung geben und dazu beitragen, dass die einzelnen Transformationsschritte am übergeordneten Ziel „gesunde Menschen auf einem gesunden Planeten“ ausgerichtet werden. Im Zentrum dieses Konzepts steht die wechselseitige Abhängigkeit zwischen dem Zustand der natürlichen Umwelt und der menschlichen Gesundheit [12]. Menschliche Gesundheit und Wohlergehen hängen unmittelbar mit globalen und lokalen Umweltveränderungen zusammen. Es geht also nicht allein um Umwelt- und Klimaschutz, sondern um den Erhalt der natürlichen Lebensgrundlagen und damit das Überleben der menschlichen Zivilisation.

Das Konzept greift außerdem zukunfts- und gerechtigkeitsbezogene Aspekte auf, indem es die Gesundheit zukünftiger Generationen als Zielgröße und gegenwärtige Ungleichheiten als Ursachen für Gesundheit und Krankheit berücksichtigt [13]. Planetare Gesundheit ist darüber hinaus eng mit den Zielen für nachhaltige Entwicklung (*Sustainable Development Goals*) der Vereinten Nationen verknüpft [14], die als eine Übersetzung des theoretischen Konzepts in konkrete, praktisch umsetzbare Ziele verstanden werden können. Die Prinzipien und Wertvorstellungen, die planetare Gesundheit auszeichnen, spiegeln sich in den grundsätzlichen Maximen der Ziele für nachhaltige Entwicklung wider, zu deren Umsetzung sich auch Deutschland mit der Neuauflage der Deutschen Nachhaltigkeitsstrategie 2017 sowie im Koalitionsvertrag 2018 verpflichtet hat [15, 16].

Wir skizzieren im Folgenden, inwiefern Angehörige der Gesundheitsberufe eine besondere Rolle für gesellschaftliche Transformation im Sinne der Ziele für nachhaltige Entwicklung spielen können und müssen. Wir diskutieren Kernherausforderungen für die Lehre zu planetarer Gesundheit und beschreiben ausgewählte Aspekte einer flächendeckenden Institutionalisierung einschlägiger Lehr- und Lernformate in der Aus‑, Fort- und Weiterbildung der Gesundheitsberufe.

## Angehörige der Gesundheitsberufe als Schlüsselfiguren transformativen Wandels

Angehörige der Gesundheitsberufe wie Mediziner*innen, Pflegende oder Psycho‑, Physio- und Ergotherapeut*innen sowie Fachkräfte im Öffentlichen Gesundheitsdienst haben das Potenzial, zu Schlüsselfiguren transformativen Wandels zu werden. Sie tragen eine besondere Verantwortung für das Wohlergehen ihrer Patient*innen sowie der gesamten Bevölkerung und sind dem Grundsatz des Nichtschadens („primum non nocere“) verpflichtet. Zudem findet sich in §1 der Musterberufsordnung für Ärzt*innen in Deutschland die Verpflichtung zur Erhaltung der natürlichen Lebensgrundlagen [17]. Diese ethische Handlungsmaxime teilen Ärzt*innen mit allen anderen Angehörigen der Gesundheitsberufe. In Zeiten der Klima- und Nachhaltigkeitskrise haben Fachkräfte des Gesundheitswesens durch dieses gemeinsame Ethos eine besondere Verantwortung, Transformationsbedarfe zu identifizieren und proaktiv Wandel anzustoßen.

Zudem sind Angehörige der Gesundheitsberufe durch das ihnen entgegengebrachte Vertrauen [18] in einer günstigen Position, auf unterschiedlichen Ebenen der Gesellschaft transformativen Wandel zu initiieren und aktiv mitzugestalten. Dies beginnt bei der Patient*innenversorgung durch Förderung nachhaltiger Verhaltensweisen mit positiven Gesundheitseffekten (*co-benefits*), wie zum Beispiel die Umstellung auf eine vorwiegend pflanzenbasierte Ernährung [19], und reicht bis zur Beratung zu gesundheits- und nachhaltigkeitsbezogenen Aspekten in politischen Prozessen.

Durch interdisziplinäre Zusammenarbeit und synergistisches Handeln können Angehörige der Gesundheitsberufe somit zur Transformation in allen gesellschaftlichen Sektoren beitragen.

## Zentrale Herausforderungen auf dem Weg zur planetaren Gesundheit

Um ein weiteres Voranschreiten der Klima- und Nachhaltigkeitskrise zu verhindern, müssen 3 zentrale Herausforderungen (*challenges*) adressiert werden. Diese Herausforderungen betreffen, in Anlehnung an Whitmee et al. [12]:

1. die *Vorstellungskraft *in Bezug auf eine Vision von Gesundheit und Wohlergehen für alle Menschen innerhalb planetarer Belastungsgrenzen, also ein Leben im Einklang mit der Natur (*imagination challenge*),
2. das *Wissen* um die Zusammenhänge zwischen der Klima- und Nachhaltigkeitskrise und menschlicher Gesundheit (*knowledge challenge*) und
3. die *Umsetzung* der Maßnahmen, die für die Sicherstellung umfassender Gesundheit und Wohlergehen innerhalb planetarer Belastungsgrenzen notwendig sind (*implementation challenge*).

Die *Herausforderung hinsichtlich der Vorstellungskraft *bezieht sich auf die Fähigkeit, Visionen einer Gesellschaft zu entwickeln, in der ein gesundes und nachhaltiges Leben für alle Menschen innerhalb planetarer Belastungsgrenzen sichergestellt ist. Diese Visionen dürfen nicht ausschließlich auf zukünftigem technologischem Wandel fußen, sondern müssen in erster Linie fundamentale ökologische, soziale und ökonomische Determinanten menschlicher Gesundheit adressieren. Die Lehre zu planetarer Gesundheit sollte diese Herausforderung aufgreifen, indem sie Weltbilder und Wertvorstellungen, inklusive ihrer ethischen Implikationen, kritisch reflektiert und neu bewertet [20]. Angehörige der Gesundheitsberufe sollten bei der Entwicklung positiver Zukunftsvisionen begleitet werden sowie Konzepte und Kompetenzen erlernen, mit denen sie diese Visionen auch im beruflichen und privaten Umfeld anregen und begleiten können. Die Fähigkeit, sich eine Welt umfassender und dauerhafter Gesundheit für alle Menschen innerhalb planetarer Belastungsgrenzen und die dafür notwendigen Rahmenbedingungen vorzustellen, ist der erste Schritt zur Realisierung planetarer Gesundheit.

Die *Herausforderung hinsichtlich des Wissens* bezieht sich einerseits auf den Umstand, dass unser Verständnis der Zusammenhänge zwischen Umweltveränderungen und menschlicher Gesundheit zum Teil noch lückenhaft ist. Andererseits ist das vorhandene Wissen den gesellschaftlichen Entscheidungsträger*innen häufig nur unzureichend zugänglich. Für die Lehre zur planetaren Gesundheit bedeutet dies, dass Angehörige der Gesundheitsberufe bei der Wissensaneignung und der kritischen Reflexion vorhandener Wissenslücken begleitet werden sollten. Sie müssen insbesondere in der Lage sein, die Auswirkungen globaler, regionaler und lokaler Umweltveränderungen auf die Gesundheit zu analysieren und hinsichtlich ihrer Tragweite einzuordnen. Zudem sollten sie Kompetenzen entwickeln, um dieses Wissen auch effektiv an Entscheidungsträger*innen kommunizieren zu können.

Die *Herausforderung hinsichtlich der Umsetzung* beschreibt die Schwierigkeit, vom Wissen ins Handeln zu kommen. Der Lückenschluss zwischen dem Wissen um die negativen Auswirkungen der Klima- und Nachhaltigkeitskrise und dem aktiven Anstoßen von Transformationsprozessen in Richtung planetarer Gesundheit ist eine Aufgabe, die auf individueller, institutioneller und gesamtgesellschaftlicher Ebene gelöst werden muss. Damit Fachkräfte im Gesundheitswesen aktiv dazu beitragen können, sollten ihre Rollen als Initiator*innen und Gestalter*innen von Transformationsprozessen (*change agents*) in einschlägigen Lehr- und Lernformaten reflektiert und gestärkt werden. Dazu ist ebenfalls die Entwicklung praktischer Kompetenzen wie effektiver Gesprächsführung notwendig.

Derzeit steht v. a. die Wissensvermittlung zu den Auswirkungen der Klima- und Nachhaltigkeitskrise im Fokus einschlägiger Lehr- und Lernformate [20]. Damit Angehörige der Gesundheitsberufe in der Aus‑, Fort- und Weiterbildung in ihrer Rolle als aktive Gestalter*innen von Transformationsprozessen gestärkt werden, ist es jedoch notwendig, dass auch die Herausforderungen hinsichtlich der Vorstellungskraft und der Umsetzung verstärkt adressiert werden. Ziele der Lehre zu planetarer Gesundheit sollten damit gleichermaßen die Wissensaneignung, die Entwicklung praktischer Kompetenzen und die kritische Reflexion von Werten und Haltungen sein – jeweils unterstützt durch angepasste didaktische Konzepte [20]. Hierfür sind sowohl inhaltlich-didaktische als auch institutionelle Reformen notwendig, die transformatives und systembasiertes Lernen und Lehren zum Ziel haben [21].

## Lehre zu planetarer Gesundheit im Gesundheitswesen

International findet die Lehre zu planetarer Gesundheit zunehmend Eingang in Aus‑, Fort- und Weiterbildungscurricula. Die frei zugängliche Datenbank der Planetary Health Alliance, einem Konsortium aus internationalen Universitäten, Nichtregierungsorganisationen, Forschungsinstituten und Regierungsstellen, zählte im November 2020 etwa 120 einschlägige Lehr- und Lernangebote [22]. Neben der Umsetzung konkreter Lehr- und Lernangebote werden beispielsweise in Kanada aktuell kompetenzorientierte Lernziele zur planetaren Gesundheit für medizinische Curricula entwickelt [23].

Während planetare Gesundheit bisher kaum in der Aus‑, Fort- und Weiterbildung des deutschen Gesundheitswesens abgebildet war, entstehen seit Kurzem auch hierzulande einschlägige Lehr- und Lernformate. Diese reichen von studentischen Initiativen über Wahlfächer (z. B. das Projekttutorium *Planetary Health* an der Humboldt-Universität zu Berlin, das Wahlfach *Klimakrise und Gesundheit* an der Medizinischen Fakultät Mannheim oder das Wahlfach *Planetare Gesundheit* an der Medizinischen Fakultät Würzburg) und Ringvorlesungen (z. B. die *Ringvorlesung Umwelt* an der Technischen Universität München oder die *Ringvorlesung Planetary Health* an der Ludwig-Maximilians-Universität München) bis zu standortübergreifenden Formaten wie die im Sommersemester 2020 virtuell abgehaltene *Planetary Health Academy* der Deutschen Allianz Klimawandel und Gesundheit (KLUG e. V.).

Der Erfolg dieser neu etablierten Angebote (z. B. über 3000 Anmeldungen für die *Planetary Health Academy*) zeigt, dass sie eine in Deutschland bis dato bestehende Lücke im Lehrangebot füllen. Nachdem die meisten Lehr- und Lernformate bisher im Umfeld medizinischer Fakultäten entstanden sind, sollten im nächsten Schritt weitere Angebote für alle Stufen der Ausbildung und alle weiteren Disziplinen des Gesundheitswesens, wie Zahnmedizin, Pflege, Therapiedisziplinen oder Public Health, etabliert werden. Dabei sind je nach fachspezifischen Anforderungen und lokalen Gegebenheiten verschiedene Lehr- und Lernformate vorstellbar, die in unterschiedlichem Ausmaß bei der Wissensaneignung, der Reflexion von Werten und Haltungen und bei der Entwicklung transformativer Kompetenzen unterstützen. Einige Beispiele für curriculare und extracurriculare Lehr- und Lernformate sind in Tab. 1 zusammengefasst. Tab. 2 gibt exemplarisch einen Überblick über das Wahlfach *Klimakrise und Gesundheit*, das seit September 2020 an der Medizinischen Fakultät Mannheim angeboten wird.

**Table 1:** 

Wahlfächer zu planetarer Gesundheit
Aufnahme von Inhalten zu planetarer Gesundheit in Pflichtcurricula
Vertiefungsangebote, die Studierenden und Auszubildenden eine individuelle Profilbildung im Bereich planetare Gesundheit ermöglichen (z. B. als Neigungsorientierung)
Transdisziplinäre Lehr- und Lernformate, die den Austausch zwischen Studierenden, Auszubildenden und Praxispartner*innen (z. B. Öffentlicher Gesundheitsdienst, Stadtplaner*innen) fördern und ggf. Praktika außerhalb des klinischen Versorgungssystems erlauben
Studiengangs- bzw. fakultätsübergreifende Angebote (z. B. Ringvorlesungen)
Förderung studentischer Initiativen (z. B. Arbeitsgruppen zu planetarer Gesundheit), ggf. mit Anrechnung der Teilnahme als Prüfungsleistung

**Table 2:** 

Zeitraum	Inhalt	Format/Methode
*Blockkurs (3 Tage, jeweils in der letzten Woche der Semesterferien)*		
Tag 1, Vormittag	Grundlagen zur Klimakrise	Input durch Lehrende
	Darstellung des Paris-kompatiblen deutschen CO_2_-Budgets und seiner Allokation über die Zeit	Verkürztes Planspiel in Kleingruppen
	Grundlegende Aspekte der Transformationsforschung (z. B. Hauptgutachten des Wissenschaftlichen Beirats Globale Umweltveränderungen, Transformationsarenen nach Schneidewind)	Input durch Lehrende
	Einführung in die planetare Gesundheit als umfassendes Gesundheitskonzept	Input durch Gastdozierende und Reflexion in Kleingruppen sowie im Plenum
Tag 1, Nachmittag	Einführung in die gesundheitlichen Auswirkungen der Klimakrise	„Think-pair-share“ und ergänzender Input durch Lehrende
	Ethische Dimensionen der Klimakrise und ihrer gesundheitlichen Auswirkungen	Kleingruppenarbeit und Input durch Lehrende
Tag 2, Vormittag	Einführung in praktische Aspekte transformativen Handelns	Input durch Gastdozierende
	Beispiele zur praktischen Umsetzung von Transformationsprozessen in Kliniken und Kommunen	Input durch Praxispartner*innen und anschließende Diskussion
Tag 2, Nachmittag	Einführung in studentische Praxisprojekte aus dem Bereich *Klima und Gesundheit* als Prüfungsleistung	Input durch Lehrende
	Entwicklung erster Ideen zu Praxisprojekten	Kleingruppenarbeit
Tag 3, Vormittag	Vorstellung lokaler Initiativen aus dem Bereich *Klimakrise und Gesundheit* (z. B. Health for Future)	Kurzinput durch Praxispartner*innen und Diskussion im Plenum
	Arbeit an den Praxisprojekten	Kleingruppenarbeit
Tag 3, Nachmittag	Arbeit an den Praxisprojekten	Kleingruppenarbeit
Täglich	Eingangs- und Abschlussrunde	
*Termine während des Semesters (2 Termine, Dauer: jeweils 90* *min)*		
Termin 1	Arbeit an den Praxisprojekten und Klärung offener Fragen	Kleingruppenarbeit und Diskussion im Plenum
Termin 2	Präsentation der Praxisprojekte	Präsentation durch Kleingruppen und Reflexion im Plenum
*Prüfungsleistung*		
Präsentation des Praxisprojekts als Kleingruppe im Rahmen des Wahlfachs		
Schriftliche Reflexion zum Praxisprojekt		

Parallel ist die Entwicklung von Weiter- und Fortbildungsformaten für Fachkräfte des Gesundheitswesens notwendig. Die Fortbildungspflicht für Fachärzt*innen bietet beispielsweise eine gute Grundlage, um einen bedeutenden Anteil von Mediziner*innen mit Fortbildungsangeboten zur planetaren Gesundheit zu erreichen.

Um die flächendeckende Etablierung einschlägiger Lehr- und Lernformate in der Aus‑, Fort- und Weiterbildung zu unterstützen, sollten Entwicklungsprozesse an einzelnen Standorten durch standortübergreifende Institutionalisierungsprozesse begleitet werden.

*Standortübergreifende Entwicklung und Verankerung verbindlicher Lernziele zur planetaren Gesundheit:* Gemäß den oben beschriebenen Herausforderungen hinsichtlich der Vorstellungskraft, des Wissens und der Umsetzung sollten Lernziele zur planetaren Gesundheit neben der Wissensvermittlung insbesondere Kompetenzen abdecken, die für die Initiierung und Umsetzung wirksamer Transformationsprozesse notwendig sind. Dazu wäre die zeitnahe Verankerung von verbindlichen nationalen Lernzielen zur planetaren Gesundheit für alle Gesundheitsberufe sinnvoll. Die derzeit laufende Weiterentwicklung des Nationalen Kompetenzbasierten Lernzielkatalogs Medizin (NKLM) und des Gegenstandskatalogs Medizin (GK) bietet hierzu eine gute Gelegenheit.

Dabei könnten bereits etablierte Lehrprinzipien und ein international derzeit in Entwicklung befindlicher Musterlernzielkatalog als Ausgangspunkte dienen [22, 24]. Auf Basis standortübergreifend gültiger Lernziele könnten anschließend an allen medizinischen Fakultäten vergleichbare Lehr- und Lernformate neu entwickelt bzw. bestehende Formate ergänzt werden. Ähnliche Prozesse sollten auch für andere Berufsgruppen im Gesundheitswesen angestoßen werden. Für die Zahnmedizin, Psychotherapie und Pharmazie könnte die Entwicklung bzw. Überarbeitung der spezifischen Gegenstandskataloge am Institut für medizinische und pharmazeutische Prüfungsfragen (IMPP) ein sinnvoller Ausgangspunkt sein. Hierzu wurde am IMPP im Herbst 2020 bereits eine interprofessionelle Arbeitsgruppe eingesetzt.

*Entwicklung von Mustercurricula und lernzielorientierten Prüfungsformaten:* Aufbauend auf standortübergreifenden Lernzielen könnten die Entwicklung und modellhafte Erprobung von Mustercurricula die flächendeckende Umsetzung von didaktisch hochwertigen Lehr- und Lernformaten zur planetaren Gesundheit unterstützen. Durch einen modularen Aufbau der Mustercurricula könnte sichergestellt werden, dass diese nach Abschluss der Erprobungsphase verhältnismäßig einfach auf verschiedene Ausbildungseinrichtungen mit unterschiedlichen Studien- und Prüfungsordnungen übertragbar sind. Im Sinne des *Constructive Alignment* [25], d. h. der Ausrichtung nicht nur der Lehr- und Lernmethoden und -inhalte, sondern auch der Prüfungsformate an bestehenden Lernzielen, sollten außerdem Prüfungsformate entwickelt werden, die das Erreichen der übergeordneten Lernziele auf optimale Weise unterstützen. Aktuell erproben einzelne Standorte unterschiedliche Lehr- und Lernformate. Bei Interesse an diesen Entwicklungen steht die Korrespondenzautorin dieses Artikels sehr gerne zur Verfügung.

*Kapazitätsaufbau unter Lehrenden*: Lehre zu planetarer Gesundheit, wie in diesem Beitrag skizziert, geht weit über das gegenwärtige Lehrportfolio von Dozierenden in Bildungseinrichtungen des Gesundheitswesens hinaus [26]. Insbesondere die Begleitung von Studierenden, Auszubildenden und Fachkräften bei der Entwicklung von Kompetenzen, die für die Initiierung und Umsetzung von Transformationsprozessen notwendig sind, ist anspruchsvoll und setzt spezifische didaktische Fertigkeiten voraus. Vor diesem Hintergrund sollten Angebote geschaffen werden, die den Kapazitätsaufbau unter Dozierenden unterstützen (z. B. Etablierung (hochschul‑)didaktischer Weiterbildungsangebote zum transformativen und erfahrungsbasierten Lernen). Dabei kann auch die inhaltliche und didaktische (Weiter‑)Entwicklung von Lehr- und Lernformaten unter aktiver Einbindung von Studierenden und Auszubildenden unterstützend wirken [26].

*Evaluation transformativer Lehrformate*: Da transformative Lehrformate in den meisten Institutionen neu entwickelt werden müssen, sollte die Entwicklung im Rahmen von Lehrforschungsprojekten engmaschig evaluiert werden [20]. Ein standortübergreifendes Vorgehen könnte die Sichtbarkeit und Aussagekraft dieser Evaluationsergebnisse erhöhen.

## Fazit

Die Klima- und Nachhaltigkeitskrise bedroht die menschliche Gesundheit heute und in der Zukunft. Angehörige der Gesundheitsberufe haben eine ethische Verantwortung, Gesundheit zu schützen. Durch das in sie gesetzte Vertrauen besitzen sie zudem ein besonderes Potenzial, gesellschaftliche Transformation zu initiieren und zu gestalten. Damit Angehörige der Gesundheitsberufe jedoch als aktive Gestalter*innen des Wandels zu einem gesunden und nachhaltigen Leben für alle Menschen beitragen können, muss die Aus‑, Fort- und Weiterbildung flächendeckend am Konzept der *planetaren Gesundheit* ausgerichtet werden. Die Zeit ist reif.

### Infobox 1 Konzept der planetaren Belastungsgrenzen (Planetary Boundaries)

- Beschreibt Grenzwerte für die ökologische Tragfähigkeit 9 natürlicher Systeme oder Prozesse
- Grenzwerte beruhen auf wissenschaftlichen Erkenntnissen und dem Vorsorgeprinzip
- Kipppunkte (*Tipping Points*, empirisch belegt oder postuliert) markieren irreversible und rapide Veränderungen natürlicher Systeme und Prozesse mit potenziell katastrophalen Auswirkungen auf die Lebensbedingungen auf der Erde
- Beispiel für eine Belastungsgrenze in Bezug auf die Klimakrise: CO_2_-Konzentration in der Atmosphäre (aktuell ca. 410 Parts per Million [27], postulierter Kipppunkt ≥ 450 Parts per Million [5]) führt wahrscheinlich zum Abschmelzen der Polkappen und Verlust von Permafrostböden, sodass sich die Erderwärmung exponentiell beschleunigen würde [28]
